# Influence of Smoke on Health

**Published:** 1908-12

**Authors:** 


BUFFALO MEDICAL JOURNAL.

A Monthly Review of Medicine and Surgery.

*EDITOR:*

WILLIAM WARREN POTTER, M. D.

All communications, whether of a literary or business nature, books for reviewand exchanges, should be addressed to the editor, 238 DELAWARE AVENUE, BUFFALO, N. Y.

VOL. LXIV. DECEMBER, 1908. No. 5

### Influence of Smoke on Health

UNDER this title the *New York Tribune* recently discussed editorially a question which concerns the welfare of people living in large cities in a constantly increasing degree. At the present time, Buffalo is urging the increase of its manufacturing plants, is inviting with alluring offers of advantages, additional smoke-producing factories to locate within the boundaries of this attractive city. For years, Buffalo has been one of the most delightful of summer cities, one of the most comfortable all-the-year-round cities for the residential population, and especially, has it been attractive to the summer tourist.

Of late, however, the smoke nuisance has taken on such large proportions, that the thoughtful citizen who would regard health as quite necessary to prosperity, even indispensable thereto, has begun to inquire if it may not be necessary for the municipality to adopt stringent measures to restrain the already existing volume of smoke output, as well as to prevent any further increase which may result from incoming manufacturing plants. We do not wish to be understood as against progress, as opposed to the material interests of this great and constantly growing city, but we would not have its fair name and fame as a healthful place of residence injured or abused by creating an unhealthful environment for the residential population.

In the issue of the JOURNAL for January, 1906, Dr. Dewitt C. Greene, of this city, presented the evils growing up around the smoke nuisance in a thoroughly practical and scientific fashion. We advise all interested to read it or reread it as the case may be, for it will bear repetition in the reading if it has been read but once. In this relation we invite attention to the article in *The Tribune* already referred to, and which we reproduce for the benefit of our readers:

### INFLUENCE OF SMOKE ON HEALTH.

What may be considered from the physician's point of view the most hurtful effects of soft coal smoke are pointed out in *The New York Medical Record* by Dr. John W. Wainwright, of this city. One of them is that it robs a community of the sunlight which is essential to physical wellbeing. Another is that the imperfect combustion which liberates unconsumed particles of carbon also generates poisonous gases. A third is that in order to exclude the soot from their houses many persons shut their windows, and breathe a vitiated atmosphere indoors. Dr. Wainwright does not believe that smoke exercises a germicidal influence. On the contrary, he thinks that there is some relation between the heavy mortality from tuberculosis in big towns like Manchester and Leeds, England, and the smudge with which they are continually overhung. Indirect as well as direct harm is attributed by Dr. Wainwright to the same agent. In his opinion, by depressing the spirits and causing mental irritation it aggravates the condition of those who suffer from certain diseases of the heart and nervous system.

Much mischief is also done by smoke to clothing and merchandise. A Chicago man asserts that the damage to his goods in a single year from this cause amounted to $200,000, and the statement is not absolutely incredible. Still, if it is true, what must be the losses in the whole city where he lives, and in a score of other places in the west afflicted in the same manner?

Fortunately, New York city has been bothered much less than many other large centers of population by this nuisance, but the increasing use of soft coal and carelessness in the use of it made corrective measures necessary here. In one other respect the metropolis is exceptionally favored. The enforcement of the ordinance forbidding the production of smoke in New York is undertaken by the Board of Health, which has practically unlimited authority in regard to anything which it pronounces unsanitary. Dr. Wainwright's article shows how ample is the justification for the energetic policy of Dr. Darlington, and to those cities in which the official crusade against smoke is a farce, a careful study of the charter of New York is respectfully commended.

It is quite important that this question should be dealt with intelligently before it grows to inordinate proportions, before its power for evil becomes too great, before the smoke creators become so strong as to resist successfully the more timid sanitarians.

In the city of New York, a group of influential women have been battling against the nuisance created by unnecessary noises, and they have succeeded in stopping them in a very considerable degree. Why should not the patriotic and home-loving women of Buffalo take up the consideration of the influence of smoke on health with a view to create, through an educational campaign, a public sentiment that will restrain and ultimately stamp out the unnecessary making of smoke?

